# Context Matters—Child Growth within a Constrained Socio-Economic Environment

**DOI:** 10.3390/ijerph191911944

**Published:** 2022-09-21

**Authors:** Lukhanyo H. Nyati, Leila Patel, Sadiyya Haffejee, Matshidiso Sello, Sonia Mbowa, Tania Sani, Shane A. Norris

**Affiliations:** 1 SAMRC/Wits Developmental Pathways for Health Research Unit, Department of Paediatrics, Faculty of Health Sciences, University of the Witwatersrand, 7 York Rd., Parktown, Johannesburg 2193, South Africa; 2 Centre for Social Development in Africa, Faculty of Humanities, Johannesburg Business School, University of Johannesburg, Milpark 2092, South Africa

**Keywords:** urban environment, poverty, cash transfers, child growth, child resilience, maternal depression, developing countries, South Africa

## Abstract

Communities in major cities in developing countries may experience economic vulnerability, which has detrimental consequences for maternal and child health. This study investigated individual-, household-, and community-level factors associated with child growth and resilience of early-grade learners aged 6 to 8 years. Demographic characteristics, depression scale, child wellbeing, and anthropometric measurements were collected on a sample of 162 caregiver–child pairs (children 46% female) who receive the child support grant (cash transfer programme) from five low-income urban communities in the City of Johannesburg, South Africa. Height and weight were converted to z-scores using the WHO Anthroplus software. Multiple linear regression was used to assess factors associated with child health outcomes and multi-level regression to account for community-level factors. Higher income vulnerability was associated with lower weight- and height-for-age z-scores (WAZ and HAZ). Not completing secondary schooling and higher household size were associated with lower HAZ but higher BAZ. Child male sex and caregiver with depression were associated with lower child resilience. Caregiver’s level of schooling and household size remained independent predictors of child growth, while the caregiver’s mental health status independently predicted child resilience. Thus, notwithstanding systemic constraints, there may be modifiable drivers that can help in developing targeted intervention.

## 1. Introduction

Child growth is a sensitive indicator of the social and economic conditions of society [1]. The guiding principle of the 2006 World Health Organisation (WHO) child growth standards for children from birth to 5 years of age is that “children born anywhere in the world have the potential to develop to within the same range of height and weight” given a similar environment [2]. However, there are population differences in child growth and nutritional status largely driven by differences in socio-economic status. Globally, low- and middle-income countries (LMIC) have the highest prevalence of childhood undernutrition [3] while experiencing the fastest rise in the prevalence of obesity [4]. Stunting, which is the most common form of undernutrition, is associated with long-term adverse health outcomes, poor cognitive development, and lower human capital [5].

Rising levels of urbanisation, which has been described as economically disequilibrating [6], may contribute to the persistently high levels of undernutrition in sub-Saharan Africa (SSA). Popkin suggests that urbanisation in Africa has preceded broader economic growth [7], thus increasing the vulnerability of poorer urban dwellers and reinforcing the levels of inequality and poverty [8]. A study by Cameron et al. in South Africa found that urban children from average socio-economic status (SES) homes were consistently smaller than rural and well-off urban children [9]. It has been shown that recent migrants to the largest urban area in South Africa (Soweto, Johannesburg) experience greater vulnerability compared to long-term residents due to less access to basic services and household amenities and later onset of schooling [10].

**Figure 1:**
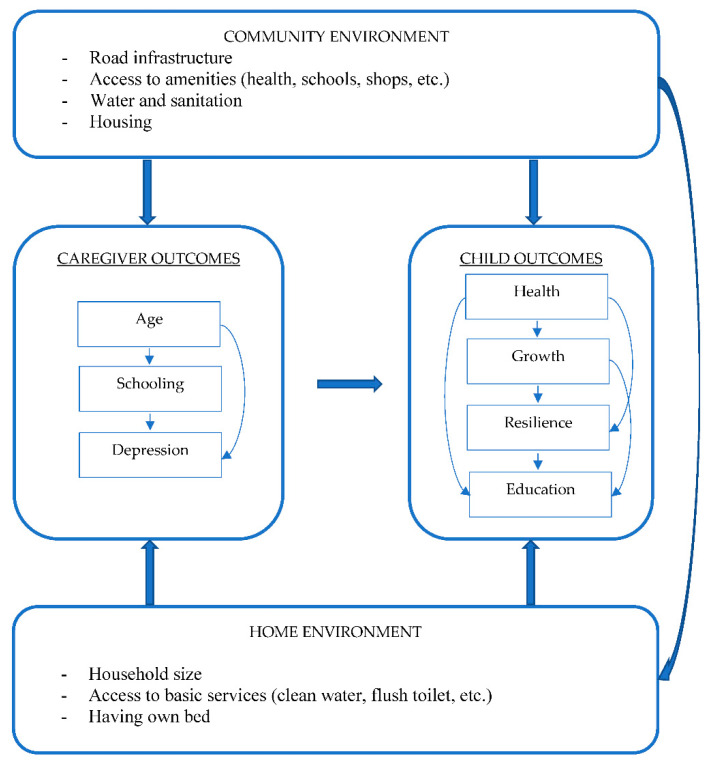
Proposed conceptual framework to demonstrate the potential relationship between individual-, household-, and community-level factors.

Although broader macroeconomic factors may drive this vulnerability, local interventions have shown efficacy in improving child growth and development in low-income settings. Nutritional programmes targeted on children, and educational programmes to empower caregivers have yielded positive benefit for child growth [11]. The Community of Practice (CoP) for Social Systems Strengthening to improve child wellbeing outcomes is a multi-disciplinary programme that seeks to investigate appropriate cross-sectoral interventions to step up child wellbeing outcomes and delivering these across the health, education, and social welfare sectors as well as achieving social sector systems strengthening to improve child wellbeing in urban communities. The economic vulnerability in poor urban communities may have been exacerbated by the coronavirus disease 2019 (COVID-19) pandemic, and the data for the first wave of the CoP were collected in 2020, during the height of the COVID-19 pandemic. The aims of the current study were to present baseline findings from the CoP, to (i) describe the growth of children from five low-income urban settings, and to (ii) assess the association of individual- and household-level factors with child growth and mental health status, accounting for community-level variance. The conceptual framework in Figure 1, which represents the hypothesis tested in this study, is based on the ecological theoretical framework for health promotion [12].

## 2. Materials and Methods

### 2.1. Participants and Context

This was a baseline study of the Community of Practice (CoP) intervention, which is a multi- and transdisciplinary initiative involving collaboration between researchers and practitioners across different sub-fields. Data were collected between October and December 2020 on 162 caregivers and children in the foundation years of schooling, Grades R and Grade 1, who are recipients of the child support grant. Participants were selected from five conveniently selected schools (Table 1) in areas defined (by CoJ) as critically poor in the City of Johannesburg (CoJ). All the schools were no-fee-paying schools except for the one in Region F. Public schools in South Africa are divided into five quintile rankings; the lowest three quintiles are no-fee-paying schools [13].

### 2.2. Demographic Characteristics, Child Wellbeing, and Depression Scales

Demographic characteristics were collected using a standard questionnaire, while the resilience score and caregiver depression score were collected using validated questionnaires. The Centre for Epidemiological Studies’ Depression Scale (CES-D-10), shown to be valid for the South African population [14], was used to assess depressive symptomology. Psychosocial development and wellbeing were further assessed using the Child and Youth Resilience Measure (CYRM) [15].

Child wellbeing was assessed using a child wellbeing tracking tool (CWTT) developed by CoP partners and was not validated. The aim was to assess child wellbeing by including both subjective as well as objective indicators of child wellbeing. The focus was therefore on the child and his/her family as well as the systems surrounding the child. The CWTT contained six sections, namely, demographic and social profile, child wellbeing domains, parent/caregiver health and wellbeing, education and wellbeing domain, health domain, and subjective measures. The CWTT further drew on some aspects of a similar child wellbeing tool developed by United Nations Children’s Fund (UNICEF) to assess children of all age groups [16]. Both a literature review of child wellbeing and findings from various studies conducted by the respective chairs informed the domains included in the questionnaire. For each child sampled, data were collected from important role players in terms of the child’s overall wellbeing. These role players included: the caregivers, the schoolteachers, the healthcare workers, and the children themselves. The children and caregivers were interviewed by a social worker who completed the questionnaire. The teachers completed the questionnaires themselves. The nursing preceptors conducted a physical examination and completed questionnaires.

### 2.3. Physical Measurements

Height and weight were measured using standard techniques, with children in minimal clothing, by trained nursing preceptors. Height was measured to the nearest 0.1 cm, and weight was measured to the nearest 0.1 kg using standard techniques and instruments.

### 2.4. Statistical Analysis

Height and weight were converted to z-scores using the WHO Anthroplus Software (version 1.0.4) (Geneva, Switzerland), which converts anthropometric measurements to z-scores using age- and sex-matched standards from the 2006 WHO child growth standards for children from birth to 5 years and the 2007 WHO growth references for children from 5 to 19 years. Differences in categorical variables were assessed using the chi-square test. Differences in count data (total number of grants and household size) were assessed using the Mann–Whitney test or Kruskal–Wallis (with Dunn test for multiple comparisons) and presented as median and range. Differences in continuous variables were assessed using a *t*-test or one-way analysis of variance (ANOVA),with Turkey multiple comparisons test.

Factors associated with child growth parameters and resilience were assessed using hierarchical multiple linear regression analysis. Logistic regression analysis was used to assess factors associated with the education domain. Bivariate analyses were performed, and variables were added based on a *p*-value less than 0.2 or established relationships in the literature. Variables were added in blocks at child level, caregiver level, and household level. Multilevel regression analyses were used to account for community-level variation using school classification (fee-paying vs. non-fee-paying schools). All analyses were performed at the 5% significance level using RStudio (Version 1.1.383—© 2009–2017 RStudio, Inc., Boston, MA, USA).

## 3. Results

### 3.1. Differences in Demographic and Health Characteristics by Child Biological Sex and Income Vulnerability

There were no sex differences in maternal and child health outcomes and demographic characteristics (Table 2) except for household size, which had a higher spread for families of male than female children (*p* < 0.05). Both boys and girls were shorter than the WHO growth references (−0.70 and −0.55 standard deviation scores (SDS), respectively). The weights of girls approached those of the WHO references, while boys were lighter (−0.36 SDS). Children from both sexes had higher BMI than the WHO reference. Further analyses were performed with both sexes combined. In Table 3, higher income vulnerability was associated with lower WAZ (*p* < 0.05) and HAZ (*p* < 0.01).

### 3.2. Differences in Demographic and Health Characteristics by School Location

There were significant differences in caregiver age (*p* < 0.05), household size (*p* < 0.01), and number of social grants per family (*p* < 0.001) between school locations (Table 4). Families from Malvern had the least concern with access to basic services, with only 9.4% showing some concern, compared to 41.7% in Ivory Park and 31.8% in Alexandra.

A combined prevalence of 96% of children from Doornkop had some or major concern for the child health domain compared to 69.7% in Ivory Park and 81.6% in Malvern. Similarly, children from Doornkop were significantly shorter but had greater BMI than children from Ivory Park, Malvern and Meadowlands, and Alexandra (*p* < 0.001). There were differences in the prevalence of depression among caregivers between locations (*p* < 0.05), with caregivers in Meadowlands having the highest prevalence (76.5%), while it was lowest in Ivory Park (41.2%)

### 3.3. Factors Associated with Child Growth

Having a caregiver who has not completed secondary schooling, a higher household size, sole dependence on social support grant income, and not having a mattress or bed to sleep on were associated with lower HAZ (Table 5). After accounting for school-level variance (fee- or non-fee-paying school), the effects were attenuated and remained significant only at the 10% significance level (i.e., *p* < 0.10). Household-level factors and maternal education accounted for ~18% of the variance in HAZ. Caregiver’s schooling was independently associated with BAZ (*p* < 0.05) even after accounting for school-level variance. The association with additional home income was attenuated after accounting for school-level variance and only became significant at the 10% significance level.

### 3.4. Factors Associated with Child Resilience

Child’s biological male gender (*p* < 0.05), having a caregiver with depression (*p* < 0.01), and living in a home with additional income to social support grants (*p* < 0.05) were associated with significantly lower child resilience score (Table 6).

### 3.5. Factors Associated with Child Education Domain

Having overweight or obesity, higher resilience, and being unemployed were associated with lower odds of having some or major concern in the education domain (*p* < 0.05) (Table 7). This can be attributed to higher odds of a caregiver’s perception of their child progressing with their schoolwork. Children with overweight/obesity were 3.4 (CI: 1.05–15.5) times more likely to be perceived to be progressing with their schoolwork than children with no overweight/obesity.

## 4. Discussion

This study presents the baseline findings from the CoP, which assessed factors associated with child growth and mental health status. The ecological framework adopted for this study assessed factors at the individual and household levels while accounting for community level variation. We found significant differences in household size, access to basic services, levels of maternal depression, child health domain, and growth outcomes between the five study locations. Children from Malvern were taller and lived in households with lower household size. Notwithstanding, completing secondary/tertiary schooling by the caregiver, having additional income in the household, and having a bed or mattress for the child were associated with higher HAZ (~2.5cm increase in height) even after adjustment for household size. The better outcomes observed among participants from Malvern can be attributed to access to better amenities (e.g., health facilities) among residents living closer to the city. Settlements on the periphery of the city were less developed due to historic discriminative laws in South Africa under the apartheid system [17]. The type of dwelling is a significant predictor of food insecurity [18]. 

The caregiver’s level of schooling and mental health status are key elements for building the capacity to provide nurturing care. The caregiver’s level of schooling was positively associated with the child’s physical growth, and this relationship was independent of household size and access to resources. Studies assessing the impact of the child support grant (CSG) have shown that girls who had a caregiver with minimum grade 8 schooling had significantly higher HAZ than those with a caregiver with lower schooling [19]. All participants in the current study were recipients of the CSG, which is a cash transfer (CT) programme (R 440~USD 30 per month) to children under the age of 18 years from low-income households in South Africa. The CSG has been shown to be effective in reducing child hunger [20] and improving child nutritional status [21] and school enrolment [22].

High levels of unemployment (~34%) in South Africa have contributed to increasing levels of social dependency. Between 2001 and 2017, an increase of over 300% was observed in the number of social grant recipients compared to a 29% increase in the number of employed individuals [23]. In the current study, 65% of the caregivers were unemployed, and 35% of the households were solely dependent on a government social grant, with some households receiving multiple grants. Although a small amount, the CSG has been reported to offer dignity and unlock other channels for generating additional income [24]. In addition to providing low amounts, a general criticism of CT programmes globally has been the lack of linkages to training programmes for livelihood skills development as a package for poverty relief in low-income settings [25]. Attaching conditions to cash transfer has been shown to contribute to more long-term positive outcomes for schooling and health compared to unconditional transfers [26]. Thus, the mechanism for linking the CSG in South Africa to skill development programmes warrants further investigation.

In a review by Ruel and colleagues, conditional cash transfers are suggested to increase access to preventive and curative health services [27], which may improve child health outcomes. In the current study, the caregiver’s mental health status was a significant predictor for the child’s psychosocial development. Being a boy child and having a caregiver with depression were associated with a lower child resilience score. The concept of male susceptibility to stress may explain the sex difference in resilience score [28]. Data from South Africa show that black males experience greater disadvantage in physical growth and maturity status than black females despite being exposed to the same growth-limiting environment [29,30]. With regards to the caregiver’s mental health status, a decreased capacity to provide nurturing care may explain the lower resilience score in children of caregivers with depression. It has been shown that depression can disrupt the caregiver–child interaction due to self-preoccupation and a negative mood [31]. This disruption may also be present in environment experiencing chronic stress due to poverty and other environmental challenges [32]. Investigating factors associated with childcare in poor communities, especially in developing countries, is a key priority for the WHO [32]. 

## 5. Conclusions

In conclusion, we observed inequalities in health among caregivers and children from low-income settings, highlighting the importance of community environment in determining health status. Caregiver’s level of schooling and home living conditions remained independent predictors of child growth, while the caregiver’s mental health status independently predicted child resilience. Providing caregivers with skills to generate additional income to supplement the CSG in low-income households may contribute to better child health outcomes. The findings from this study highlight the need for improving community health services for mental health, which are critical for providing support to caregivers, and this may offset intergenerational transmission of poor psychosocial development. Thus, notwithstanding systemic constraints, there may be modifiable drivers at the community level that can help in developing targeted intervention.

## Figures and Tables

**Table 1:** List of areas where schools were selected.

Region, Ward, and Area Name 1
Region D: Meadowlands Zone 3, Ward 42
Region A: Ivory Park, Ward 77
Region F: Malvern, Ward 65
Region C: Doornkop, Ward 50
Region E: Alexandra, Ward 109

**Table 2:** Sex differences in demographic characteristics and child growth outcomes.

	Female	Male	*p*-Value
N	75	86	
Age of caregiver	35.4 (8.7)	35.8 (9.1)	0.759
Household size	5.0 (2–12)	5.0 (3–20)	0.024
Social support grants	2.0 (0–9)	3.0 (0–10)	0.923
Education level			
NSC or tertiary	31 (41.3)	30 (35.3)	0.432
Up to secondary	44 (58.7)	55 (64.7)	
Employment status			
Some employment	25 (33.3)	31 (36.0)	0.718
Unemployed	50 (66.7)	55 (64.0)	
Caregiver mental health			
No depression	34 (47.2)	32 (45.1)	0.796
With depression	38 (52.8)	39 (54.9)	
Food security domain ^δ^			
No concern	42 (80.8)	45 (76.3)	0.566
Some or major concern	10 (19.2)	14 (23.7)	
Child health domain			
No concern	15 (21.4)	9 (11.5)	0.177
Some concern	30 (42.9)	32 (41.0)	
Major concern	25 (35.7)	37 (47.4)	
Living conditions domain ^φ^			
No concern	60 (81.1)	56 (70.0)	0.111
Some concern	14 (18.9)	24 (30.0)	
Age of the child	6.5 (0.7)	6.4 (0.7)	0.558
Child resilience score	44.1 (4.4)	43.2 (4.1)	0.185
Weight-for-age z-score	−0.08 (1.11)	−0.36 (1.38)	0.167
Height-for-age z-score	−0.55 (1.28)	−0.70 (1.42)	0.501
BMI-for-age z-score	0.32 (1.21)	0.14 (1.49)	0.401

^δ^ Some and major concern combined due to low response. ^φ^ There were no responses for major concern

**Table 3:** The effect of the income vulnerability (index defined by dependence on grants and high household size) on demographic characteristics and child growth outcomes.

	Lower Vulnerability	Higher Vulnerability	*p*-Value
N	110	48	
Age of caregiver	35 (7.7)	36 (9.9)	0.554
Education level			
NSC or tertiary	42 (38.5)	19 (39.6)	0.901
Up to secondary	67 (61.5)	29 (60.4)	
Caregiver mental health			
No depression	49 (49.0)	17 (38.6)	0.250
With depression	51 (51.0)	27 (61.4)	
Food security domain			
No concern	65 (79.3)	22 (73.3)	0.504
Some or major concern	17 (20.7)	8 (26.7)	
Child health domain			
No concern	19 (18.8)	6 (12.8)	0.535
Some concern	43 (42.6)	19 (40.4)	
Major concern	39 (38.6)	22 (46.8)	
Living conditions domain			
No concern	79 (73.8)	38 (79.2)	0.475
Some concern	28 (26.2)	10 (20.8)	
Age of the child	6.4 (0.7)	6.4 (0.7)	0.880
Child resilience score	43.4 (4.3)	43.8 (4.1)	0.664
Weight-for-age z-score	−0.07 (1.32)	−0.54 (1.08)	0.023
Height-for-age z-score	−0.43 (1.25)	−1.13 (1.52)	0.007
BMI-for-age z-score	0.27 (1.38)	0.23 (1.3)	0.860

**Table 4:** The effect of school location on demographic characteristics and child growth outcomes.

	Alexandra	Doornkop	Ivory Park	Malvern	Meadowlands	Overall *p*-Value
N	22	26	36	32	44	
Age of caregiver	35.4 (7.6)	35.8 (12.1)	32.5 (7.9) ^i^*	35.6 (6.7)	38.3 (8.9)	0.071
Household size	5.0 (2–11) ^a^*	6.0 (3–12) ^f^***	5.0 (2–11)	4.0 (2–8) ^j^***	6.0 (3–20)	<0.001
Social support grants	2.5 (0–10)	3.0 (0–5) ^f^**	3.0 (0–9) ^h^*	2.0 (0–6) ^j^***	3.0 (0–9)	0.001
Education level						
NSC or tertiary	7 (31.8)	9 (34.6)	11 (30.6)	16 (51.6)	19 (43.2)	0.384
Up to secondary	15 (68.2)	17 (65.4)	25 (69.4)	15 (48.4)	25 (56.8)	
Caregiver mental health						
No depression	10 (52.6)	10 (43.5)	20 (58.8)	17 (53.1)	8 (23.5)	0.037
With depression	9 (47.4)	13 (56.5)	14 (41.2)	15 (46.9)	26 (76.5)	
Employment status						
Some employment	7 (31.8)	8 (30.8)	13 (36.1)	15 (46.9)	12 (27.3)	0.483
Unemployed	15 (68.2)	18 (69.2)	23 (63.9)	17 (53.1)	32 (72.7)	
Food security domain						
No concern	11 (64.7)	12 (85.7)	24 (88.9)	20 (80.0)	19 (70.4)	0.281
Some or major concern	6 (35.3)	2 (14.3)	3 (11.1)	5 (20.0)	8 (29.6)	
Child health domain						
No concern	3 (15.8)	1 (4)	10 (30.3)	6 (19.4)	4 (10.3)	0.002
Some concern	2 (10.5)	10 (40)	16 (48.5)	15 (48.4)	18 (46.2)	
Major concern	14 (73.7)	14 (56)	7 (21.2)	10 (32.3)	17 (43.6)	
Access to basic services						
No concern	15 (68.2)	20 (80.0)	21 (58.3)	29 (90.6)	30 (78.9)	0.028
Some concern	7 (31.8)	5 (20.0)	15 (41.7)	3 (9.4)	8 (21.1)	
Age of the child	6.5 (0.7)	6.3 (0.5)	6.3 (0.6)	6.5 (0.7)	6.5 (0.9)	0.499
Child resilience score	43.3 (3.7)	45.0 (2.7)	43.9 (5.1)	42.9 (4.8)	43.2 (3.8)	0.349
Weight-for-age z-score	−0.35 (1.10)	0.01 (1.30)	−0.38 (1.32)	0.25 (1.09)	−0.51 (1.32)	0.076
Height-for-age z-score	−0.67 (1.10)	−1.60 (1.69) ^e^*^, f^***^, g^**	−0.61 (1.21)	0.06 (1.10)	−0.48 (1.23)	<0.001
BMI-for-age z-score	0.08 (1.31) ^a^**	1.53 (1.13) ^e^***^, f^**^, g^***	−0.01 (1.40)	0.3 (1.00)	−0.32 (1.25)	<0.001

* *p* < 0.05; ** *p* < 0.01; *** *p* < 0.001; a = Alexandra vs. Doornkop; e = Doornkop vs. Ivory Park; f = Doornkop vs. Malvern; g = Doornkop vs. Meadowlands; h = Ivory Park vs. Malvern; i = Ivory Park vs. Meadowlands; j = Malvern vs. Meadowlands.

**Table 5:** Individual- and household-level factors that are associated with child growth outcomes with adjustment for community level factors using school classification (fee-paying or non-fee-paying).

	Height-for-Age Z-Score				BMI-for-Age Z-Score			
	Model 1	Model 2	Model 3	Model 4	Model 1	Model 2	Model 3	Model 4
Child factors								
Health domain (ref: no concern)								
Some concern	−0.19 (0.35)	−0.12 (0.36)	0.03 (0.34)	0.02 (0.33)	0.00 (0.33)	−0.06 (0.33)	−0.02 (0.34)	−0.02 (0.34)
Major concern	−0.41 (0.35)	−0.33 (0.35)	−0.11 (0.33)	−0.08 (0.34)	0.18 (0.33)	−0.22 (0.33)	0.05 (0.33)	0.05 (0.33)
Maternal factors								
Education level (ref: NSC or tertiary)								
Up to secondary		−0.40 (0.23)	−0.50 (0.24) *	−0.40 (0.24)		0.46 (0.23)*	0.50 (0.24) *	0.50 (0.24) *
Household-level factors								
Household size			−0.13 (0.06) *	−0.10 (0.06)			0.05 (0.06)	0.05 (0.06)
Additional income (ref: No)								
Yes			0.50 (0.25) *	0.44 (0.24)			−0.05 (0.24)	−0.05 (0.24)
Have bed to sleep (ref: No)								
Yes			0.84 (0.41) *	0.79 (0.40)			−0.41 (0.40)	−0.41 (0.40)
Home protects from rain (ref: No)								
Yes			−0.62 (0.40) *	−0.57 (0.41)			0.66 (0.40)	0.66 (0.40)
Access to toilet (ref: No)								
Yes			−0.54 (0.37)	−0.71 (0.38)			−0.21 (0.37)	−0.21 (0.37)
R^2^	0.016	0.043	0.179		0.002	0.032	0.039	
F-test		2.52	4.11 **			3.83	0.228	

* *p* < 0.05; ** *p* < 0.01; Model 1: child health domain; Model 2: model 1 + caregiver schooling level; Model 3: model 2 + (household size, having bed, home protecting from rain, access to toilet); Model 4: model 3 with random effects for school classification.

**Table 6:** Individual- and household-level factors that are associated with child resilience with adjustment for community level factors using school classification (fee-paying or non-fee-paying).

	Child Resilience Score		
	Model 1	Model 2	Model 3
Child factors			
Child sex (ref: Female)			
Male	−1.51 (0.74) *	−1.63 (0.75) *	−1.80 (0.74) *
Maternal factors			
Caregiver depression (ref: none)			
With depression	−1.97 (0.75) **	−2.15 (0.75) **	−2.26 (0.74) **
Household-level factors			
Household size		−0.22 (0.18)	−0.28 (0.18)
Additional income (ref: No)			
Yes		−1.57 (0.77) *	−1.42 (0.77)
R^2^	0.086	0.122	
F-test		2.39	

* *p* < 0.05; ** *p* < 0.01. Model 1: child sex and caregiver depression. Model 2: model + caregiver employment status. Model 3: model 2 with random effects for school classification.

**Table 7:** Individual- and household-level factors that are associated with the child education domain with adjustment for community level factors using school classification (fee-paying or non-fee-paying).

	Education Domain		Progress Item		Afraid Item	
	Model 1	Model 2	Model 1	Model 2	Model 1	Model 2
Child factors						
Overweight and obesity (ref: none) With overweight and obesity	0.19 (0.03–0.76) *	0.25 (0.06–0.84) *	3.42 (1.05–15.51)	3.37 (1.03–15.3)	1.27 (0.62–2.63)	1.31 (0.63–2.74)
Child resilience	0.89 (0.79–1.01)	0.89 (0.79–0.99) *	1.12 (1–1.26) *	1.13 (1.01–1.27) *	0.93 (0.86–1.01)	0.92 (0.85–1)
Household factors						
Employment (ref: some employment) Unemployed	0.31 (0.11–0.89) *	0.31 (0.11–0.79) *	2.56 (0.97–6.9)	2.54 (0.97–6.85)	0.95 (0.48–1.91)	0.93 (0.46–1.88)

* *p* < 0.05; Model 1: child overweight status and resilience, and caregiver employment status; Model 2: model 1 with random effects for school classification.

## Data Availability

The deidentified data that were used for generating the results in this manuscript can be made available upon request.

## References

[1] Tanner J.M. (1987). Growth as a Mirror of the Condition of Society: Secular Trends and Class Distinctions. Pediatr. Int..

[2] Kmietowicz Z. (2006). Children worldwide can grow to the same height, says WHO. BMJ.

[3] de Onis M., Blössner M., Borghi E. (2011). Prevalence and trends of stunting among pre-school children, 1990–2020. Public Health Nutr..

[4] Ng M., Fleming T., Robinson M., Thomson B., Graetz N., Margono C., Mullany E.C., Biryukov S., Abbafati C., Abera S.F. (2014). Global, regional, and national prevalence of overweight and obesity in children and adults during 1980–2013: A systematic analysis for the Global Burden of Disease Study 2013. Lancet.

[5] Stein A.D., Wang M., Martorell R., Norris S.A., Adair L.S., Bas I., Sachdev H.S., Bhargava S.K., Fall C.H., Gigante D.P. (2009). Growth patterns in early childhood and final attained stature: Data from five birth cohorts from low- and middle-income countries. Am. J. Hum. Biol..

[6] Greyling L., Mears R. (2014). Demographic characteristics of Soweto: A comparison of 1993 and 2008. Financ. Bank..

[7] Popkin B.M. (2006). Global nutrition dynamics: The world is shifting rapidly toward a diet linked with noncommunicable diseases. Am. J. Clin. Nutr..

[8] (2006). South African State of the Cities Report 2006. Braamfontein. www.sacities.net.

[9] Cameron N., Kgamphe J.S., Leschner K.F., Farrant P.J. (1992). Urban-rural differences in the growth of South African black children. Ann. Hum. Biol..

[10] Richter L.M., Norris S.A., Swart T.M., Ginsburg C. (2006). In-migration and living conditions of young adolescents in Greater Johannesburg, South Africa. Soc. Dyn..

[11] Rahman A., Lovel H., Bunn J., Iqbal Z., Harrington R. (2014). Mothers mental health & infant growth—A case–control study from Rawalpindi; Pakistan. Child Care Health Dev..

[12] National Cancer Institute (2005). Theory at a Glance: A Guide for Health Promotion Practice.

[13] Ogbonnaya U.I., Awuah F.K. (2019). Quintile ranking of schools in South Africa and learners’ achievement in probability. Stat. Educ. Res. J..

[14] Baron E.C., Davies T., Lund C. (2017). Validation of the 10-item Centre for Epidemiological Studies Depression Scale (CES-D-10) in Zulu, Xhosa and Afrikaans populations in South Africa. BMC Psychiatry.

[15] Ungar M., Liebenberg L. (2011). Assessing Resilience Across Cultures Using Mixed Methods: Construction of the Child and Youth Resilience Measure. J. Mix. Methods Res..

[16] Patel L., Pillay J., Henning E., Telukdarie A., Norris S., Graham L., Haffejee S., Sani T., Ntshingila N., Du-Plessis Faurie A. (2021). Community of Practice for Social Systems Strengthening to Improve Child Well-Being Outcomes Findings from Wave 1: Tracking Child Wellbeing of Early Grade Learners and Their Families. https://www.unicef.org.

[17] Strauss M. (2019). A historical exposition of spatial injustice and segregated urban settlement in South Africa. Fundamina.

[18] Jonah C.M.P., May J.D. (2020). The nexus between urbanization and food insecurity in South Africa: Does the type of dwelling matter?. Int. J. Urban Sustain. Dev..

[19] Devereux S., Waidler J. (2017). Why Does Malnutrition Persist in South Africa Despite Social Grants?.

[20] Samson M., Heinrich C., Williams M., Kaniki S., Muzondo T., Mac Quene K., Van Niekerk I. (2008). Quantitative Analysis of the Impact of the Child Support Grant. www.epri.org.za.

[21] Aguero J.M., Carter M.R., Woolard I. (2006). The Impact of Unconditional Cash Transfers on Nutrition: The South African Child Support Grant.

[22] Samson M., Van I., Kenneth N., Quene M. (2010). Designing and Iimplementing Social Transfer Programmes. http://epri.org.za/wp-content/uploads/2016/07/Designing-and-Implementing-Social-Transfer-Programmes-EPRI.pdf.

[23] Ryan C. (2018). More People on Welfare than Have Jobs in SA. Moneyweb. https://www.moneyweb.co.za/news/south-africa/more-people-on-welfare-than-have-jobs-in-sa/.

[24] Zembe-Mkabile W., Surrender R., Sanders D., Jackson D., Doherty T. (2015). The experience of cash transfers in alleviating childhood poverty in South Africa: Mothers’ experiences of the Child Support Grant. Glob. Public Health.

[25] Molyneux M., Jones N., Samuels F. (2016). Can cash transfer programmes have “transformative” effects?. J. Dev. Stud..

[26] Baird S., McIntosh C., Özler B. (2011). Cash or Condition? Evidence from a Cash Transfer Experiment. Q. J. Econ..

[27] Ruel M.T., Alderman H., Maternal and Child Nutrition Study Group (2013). Nutrition-sensitive interventions and programmes: How can they help to accelerate progress in improving maternal and child nutrition?. Lancet.

[28] Stinson S. (1985). Sex differences in environmental sensitivity during growth and development. Am. J. Phys. Anthr..

[29] Nyati L.H., Pettifor J.M., Ong K.K., Norris S.A. (2020). Adolescent growth and BMI and their associations with early childhood growth in an urban South African cohort. Am. J. Hum. Biol..

[30] Nyati L.H., Pettifor J.M., Norris S.A. (2019). The prevalence of malnutrition and growth percentiles for urban South African children. BMC Public Health.

[31] Santona A., Tagini A., Sarracino D., De Carli P., Pace C.S., Parolin L., Terrone G. (2015). Maternal depression and attachment: The evaluation of mother–child interactions during feeding practice. Front. Psychol..

[32] Department of Child and Adolescent Health and Development (2004). The Importance of Caregiver-Child Interactions for the Survival and Healthy Development of Young Children: A Review. http://apps.who.int.

